# Entropy-Based Video Steganalysis of Motion Vectors

**DOI:** 10.3390/e20040244

**Published:** 2018-04-02

**Authors:** Elaheh Sadat Sadat, Karim Faez, Mohsen Saffari Pour

**Affiliations:** 1 Electrical Engineering Department, Amirkabir University of Technology, Tehran 15875-4413, Iran; 2 Department of Mechanical Engineering, Sharif University of Technology, Tehran 1458889694, Iran; mohsensp@mech.sharif.ir or; 3 KTH Royal Institute of Technology, 11428 Stockholm, Sweden

**Keywords:** motion vector, steganalysis, entropy, motion estimation, steganography

## Abstract

In this paper, a new method is proposed for motion vector steganalysis using the entropy value and its combination with the features of the optimized motion vector. In this method, the entropy of blocks is calculated to determine their texture and the precision of their motion vectors. Then, by using a fuzzy cluster, the blocks are clustered into the blocks with high and low texture, while the membership function of each block to a high texture class indicates the texture of that block. These membership functions are used to weight the effective features that are extracted by reconstructing the motion estimation equations. Characteristics of the results indicate that the use of entropy and the irregularity of each block increases the precision of the final video classification into cover and stego classes.

## 1. Introduction

Steganography is the basis of hidden communication. In contrast to steganography methods, steganalysis methods have been developed to detect the existence of a message in digital media such as audio, image and video. In the last decades, image steganalysis has mainly been more focused due to its popularity and simplicity, while compressed video steganalysis had received partial attention [1]. Recently, with the popularity of video sharing over the Internet, compressed videos have become the best media for steganography and have provided sufficient space for hiding messages. The compressed video has various components such as motion vectors, transformed coefficients, prediction modes and partition modes which are suitable and attractive for steganography. Research shows that video traffic will make up 82 percent of all consumer Internet traffic by 2021. Consequently, video steganography and steganalysis have been growing recently. Motion Vector (MV) is more focused for video steganography because MV based steganography has high security and embedding capacity [2].

Video steganography techniques can be divided into three generations [3]. In the first-generation methods, the MV for hiding a message is selected based on the magnitude of the motion vector and the message is embedded in the least-significant bit of a motion vector [4]. These methods do not preserve the statistical properties of a motion vector and can be detectable by statistical steganalysis methods. Second-generation methods define the proper distortion function to embedding the message. The principle of these methods is to design a suitable distortion function expressing the embedding impact on motion vectors. The purposed principle of second-generation methods is to embed a higher number of hidden message bits for changing one of the motion vectors [5,6,7], on the other hand decreasing the distortion for a given payload. These approaches include the syndrome trellis code (STC) [8], the wet paper code [9] and so forth. Formerly, STC also used in image steganalysis with different distortion functions [10]. The drawbacks of the second-generation methods are the lack of optimal localization of the motion vector and are detectable with the add-or-subtract-one method (Aoso) [11]. Third-generation methods often involve changing the motion vector to areas where some of absolute differences (SAD) are locally optimal. Motion Vector Modification with Preserved Local Optimality (MVMPLO) can be called from these methods [2]. Also, the methods that change the angle of the motion vector are from this generation [12,13,14]. In this methodology, the selection of the local optimal motion vector in the area is corresponding to the message and, as a result, preserves the motion vector optimality. The detection of methods as such is difficult even with the Aoso when the bit rate is low or the message length is low. Of course, these algorithms do not guarantee that the motion vector changes to another optimal location. So, if the local optimal estimator is performed with higher precision, these algorithms can also be detected. According to the above-mentioned reason, the near perfect estimation for the local optimality method (NPE) [3] has been able to reveal this generation with the right precision. Based on the NPE method, both bit rate and distortion parameters are simultaneously used to reconstruct the Lagrange equation for motion compensation.

The main purpose of steganalysis is to determine the existence of a hidden message. Steganalysis methods in the motion vector of the video can be divided into two general categories. The first group consists of methods that extracted the statistical characteristics of the motion vector. In this group, the process of embedding the message modeled as a noise signal that is added to the vertical and horizontal components of the motion vector [15]. Moreover, such methods attempt to detect the existence of messages by examining the continuity of motion vector values in horizontal, vertical and time directions [16]. In Reference [17], the rich model contains 44,875 features, which are extracted by applying different filters to the spatio-temporal plates of motion vectors and benefit from both spatial and temporal correlations of motion vectors. This method has a very high computational complexity due to the high number of features. These steganalysis methods have low accuracy when the embedding capacity is low due to the lack of use of the SAD parameter. The second group of steganalysis includes methods that use motion vector information and motion vector optimization criteria (such as SAD, cost function, prediction error) [18,19]. The second group has higher accuracy than the first group. The reason for the higher accuracy of the second group is the extraction of more effective and more sensitive features to embedding message. The Aoso [11] with 18 features and NPE [3] with 36 features are of this category. In references [3,11], by reconstructing the motion vector estimation formula and extracting properties of the motion vectors, its corresponding SAD and their respective encoded values, attempt to detect the embedding message. The weakness of Aoso features contrasts with low-level bit rate compression. This creates features that are very close to the extracted ones from stego video. So, this methodology makes the distinction between the two categories difficult. As a result, the accuracy of detecting the existence of the message is greatly reduced. The NPE [3] method has remedied this weakness but does not consider the effects of different statistical features of the video.

In this research work, a new feature has been investigated compared to previous studies. This feature considers a special weight for each block, in which such weight adaptation has been selected from the entropy of each block. Accordingly, the paper is organized as follows: In Section 2, motion estimation and the effect of motion vector steganography is expressed in the H.264 compressed video. In Section 3, the proposed algorithm is presented using the concept of entropy, fuzzy clustering and support vector machine (SVM) classification. By using entropy, the intrinsic statistical features of different videos are considered. In Section 4, computer validation results and the comparison with the results of related works are presented and discussed. Finally, the paper is concluded in Section 5.

## 2. Motion Compensation in H.264 Compressed Video 

The latest video coding standards have inter-prediction and intra-prediction to reduce temporal and spatial redundancy, respectively. How to optimize rate and distortion (rate-distortion optimization) is a constant challenge [20,21]. In the inter-prediction modes, encoders commonly optimized rate and distortion using Lagrangian optimization techniques [22,23].

Block matching inter-frame prediction for a block in the current frame investigates the best matching region in a search area of the encoded reference. The offset from the current block to the best matched block in a reference frame is called MV and this difference is encoded to decrease statistical redundancy. Such a procedure is identified as motion estimation (ME). The goal of ME is to reduce temporal redundancy. The difference between these blocks form a residual block (or prediction error) and then the prediction error is transformed and quantized. Also, quantized coefficients of prediction error are encoded to decrease statistical redundancy (entropy encoding). In this research, the H.264/AVC encoder has been adopted because of its popularity in video coding standards and more specifically it is often chosen for video steganography [24]. The following concepts on motion H.264 compensation are necessary to be introduced further.

### 2.1. Motion Estimation Optimization

Commonly Lagrangian optimization techniques are used for rate-distortion optimization because of their simplicity and effectiveness [3]; minimizing the distortion subject to the bit rate constraint. A Lagrange multiplier handles the trade-off between distortion and bit rate. Rate-distortion optimized ME by utilizing the Lagrangian method minimizes the cost function as follows:

(1) J=D+λR

where *J* is the Lagrangian cost; D denotes the distortion; R denotes the bits needed for motion vector difference (MVD) entropy coding; *λ* is the Lagrangian multiplier, which is found experimentally as the following expressions for H.264/AVC standard [25]:

(2) $$\lambda =\sqrt{0.85\times 2^{\left(\frac{QP-12}{3}\right)}}$$

where *QP* denotes a quantization parameter defined in the H.264 standard whose values are typically 30 and can be from 0 to 51 [26]. Equations (1) and (2) show that the distortion increases with increasing the *QP* and *λ*. In the other words, Lagrangian cost controls the bits more than distortion.

Also, Equation (1) shows that for a given MV, the corresponding Lagrangian cost must be the local minimum in a neighborhood related to the motion estimation (ME) method but because of the lossy compression and *QP* effect, MVs can be non-local optimum in their neighborhood regions. In the next section describes how to use this feature for steganalysis.

#### 2.1.1. Block Size

The macro block size is 16 × 16. With the purpose of a more precise ME, the macro block (MB) is generally separated into several blocks. A MB can be divided into one block 16 × 16, two blocks 8 × 16, two blocks 16 × 8, or four blocks in the size of 8 × 8. For every block, motion estimation is performed and then it’s MVD and quantized (prediction error) PE coefficients are entropy coded and transmitted. Form Equation (1), the smaller the sized blocks lead to higher bits for transmitting MVD and less distortion. The steganalytic methods for the H.264 video must have the ability to adapt to different block sizes to extract features.

#### 2.1.2. Distortion Function

The distortion function is the measure for determining the similarity of the two blocks and finding the best match in the motion estimation process. Usually sum of absolute differences (or sum of absolute transformed differences) used as distortion function:

(3) $$SAD(S,S_{mv}^{ref})=\sum_{i}\sum_{j}\left|S(i,j)-S_{mv}^{ref}(i,j)\right|$$

(4) $$SATD(S,S_{mv}^{ref})=\sum_{i}\sum_{j}HT(\left|S(i,j)-S_{mv}^{ref}(i,j)\right|)$$

where HT is Hadamard transform

#### 2.1.3. Bits of Motion Vector Difference

Generally, the software of encoders utilizes exp-golomb coding for bit approximation in rate-distortion optimized ME to decrease computation. Thus, from the motion vector predictor of block ($mv_{}^{pred}$), the bit rate can be calculated as follows [27]:

(5) $$map(x)=\{\begin{array}{cc} 2\left|x\right| & x\leq 0\\ 2\left|x\right|-1 & x≻0 \end{array}\;\mathrm{complexity}$$

(6) $$R_{x}=2\left\lfloor \log_{2}(map(mv_{x}-mv_{x}^{pred}))\right\rfloor +1$$

(7) $$R=R_{x}+R_{y}$$

where *x* and *y* are horizontal and vertical component of motion vector and $R_{x}$ is the number of bits needed for horizontal component coding of MVD, $mv_{x}^{pred}$ denotes the horizontal component of the block’s motion vector predictor and R is the total bits required for entropy coding of MVD.

## 3. Proposed Method

Gormish and Gill [28] approximately modeled the 2D-DCT (2-dimensional-discrete cosine transform.) coefficients of PE with the Laplace distribution. In Reference [12], the quantized PE distribution was computed by *QP* and *α*, where *QP* refers to the quantization parameter and α is the parameter of the distribution and is related to the movement of objects, texture and ME method. Therefore, the texture of blocks is obtained by using the entropy value of each block in a frame can be approximated *α*. According to the ME method, the block with more texture has greater accurate motion vector. In other words, the entropy value as a criterion will be the importance of the steganalysis features of blocks. According to the previous research in Ref. [29], it has been observed that the definition of entropy was used for steganalysis of still image. However, in the proposed method, the regional entropy is used to estimate the entropy of each block. As the entropy is a measure of texture, such texture can be also implemented for image steganalysis. This feature has been previously reported in Refs. [30,31]. They reported that the texture data was extracted based on the local binary pattern (LBP) and wavelet coefficients for image steganalysis. 

**Figure 1:**
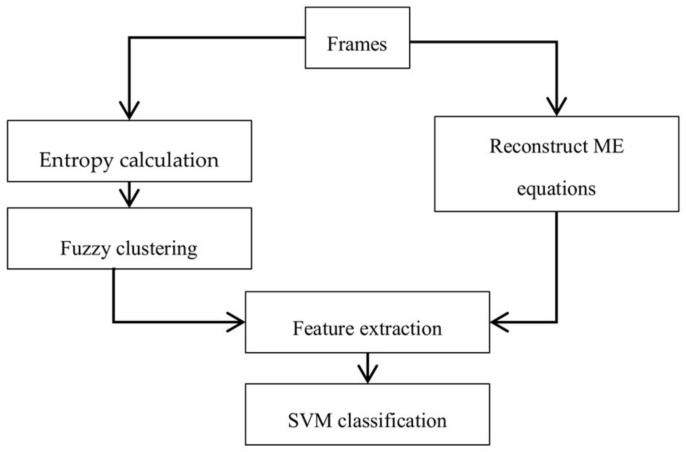
Diagram of the proposed method.

Previously, the idea of weighting the features (corresponding to probability of embedding the message) was used in image steganalysis [32]. They showed that the textural region has a higher probability for embedding a message. In the present work, by using the average entropy of pixels in the block and clustering technique, blocks can be divided into two clusters, high-textured blocks and low-textured blocks. Fuzzy clustering was used because of soft clustering which is the block that belongs to more than one cluster. The greater the entropy of the blocks, the greater its membership in the cluster with higher texture. The membership function of each block into a high entropy cluster was used to weight its feature vector to decide on the existence of a hidden message in the video. Features were extracted from properties of the reconstructed cost function and combined with the weight from the texture for every block and then the final classification is applied as a distinction between cover and stego classes. A schematic block diagram is presented to illustrate the proposed method in Figure 1.

### 3.1. Texture Measure 

**Figure 2:**
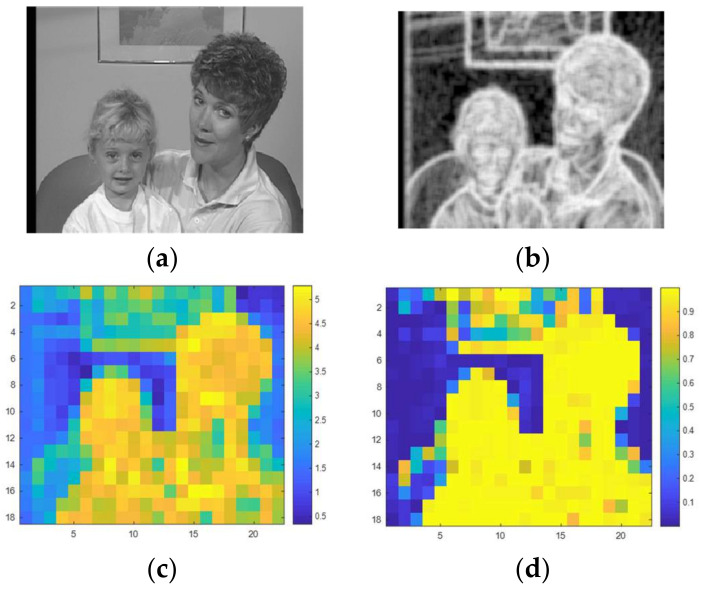
(**a**) Original frame; (**b**) Regional entropy of original frame; (**c**) Entropy of blocks of original frame and (**d**) Membership degree to high textured cluster of original frame.

Entropy is a measure of randomness that can be employed to define the texture of the image. Entropy is identified as:

(8) $$e(k)=-\sum_{i=0}^{N_{bin}}\left(p_{i}^{k}\cdot \log_{2}(p_{i}^{k})\right),$$

where $p_{i}^{k}$ contains the normalized histogram counts of the image in a 9-by-9 neighborhood around the corresponding *k*th pixel and, $N_{bin}$ refers to the number of bins of the image histogram. For regional entropy calculation for each pixel, we used the entropy value of the 9-by-9 neighborhood around the corresponding pixel in the input image. Therefore, entropy of each block is obtained by meaning of its pixels. Figure 2b,c show the entropy of the sample frame.

(9) $$E_{i}=\frac{1}{N_{pixel}}\sum_{k=1}^{N_{pixel}}e(k)$$

### 3.2. Texture Clustering

The image segmentation with statistical features is common [33]. Similarly, block clustering for the compressed video is used in this research. For clustering blocks based on texture, Fuzzy c-means (FCM) is used, that is a clustering method that permits each block to belong to numerous clusters with various degrees of membership. Such characteristics assist all blocks of frames according to their membership function to affect the final features. FCM basis is the minimization of the objective function defined as [34].

(10) $$J_{m}=\sum_{j=1}^{2}\sum_{i=1}^{N_{bolck}}\mu_{ij}^{m}(E_{i}-c_{j})$$

where $N_{bolck}$ is the number of blocks in a video, *m* is fuzzy partition matrix interpreter for managing the amount of fuzzy overlap (*m* > 1). *x_i_* is the *i*th block, *c_j_* refers to the center of the *j*th cluster and *μ_ij_* demonstrates the amount of membership of $E_{i}$ in the *j*th cluster. The membership’s degree is defined as:

(11) $$\mu_{ij}=\frac{1}{\sum_{i=1}^{N_{block}}{\left(\frac{\left\|E_{i}-c_{j}\right\|}{\left\|E_{i}-c_{k}\right\|}\right)}^{\frac{2}{m-1}}}$$

### 3.3. Feature Extraction

From Equation (1), without considering the effect of quantization, it can be concluded that each motion vector in its neighboring regions minimizes the cost function. Thus, it can be concluded that for each video, if the cost function of their motion vector is locally optimum, that video is a cover. If the video contains blocks with motion vectors that do not preserve the local optimality of the cost function, that video is a stego. By considering the effect of quantization step and lossy compression, the reconstructed frame in a decoder is not equal to the original frame in an encoder; therefore, distortion function that reconstructed in a decoder is not equal to the same function in a decoder and the cost function cannot be restructured completely. As a result, the extracted properties of the cost function for each MV must be robust to small variations of the cost function that is due to decoding. 

As mentioned in the previous section, the greater the entropy of each block, the larger the block texture and, thus, the greater the precision of its ME. So, the amount of each block’s membership in a large texture cluster can express the importance of the features of that block. A membership degree of the ith block to a cluster of high texture is obtained as:

(12) $$w_{i}=\frac{\mu_{ih}}{\sum_{k=1}^{N_{block}}\mu_{kh}},h=\arg\max\{c_{1},c_{2}\}$$

Then, for feature extraction from the reconstructed cost function, for every block, a neighborhood is defined around its MV (v=(x,y)) as N(v):

(13) $$N(v)=\{v^{\left(\Delta x,\Delta y\right)}|v^{\left(\Delta x,\Delta y\right)}=(x+\Delta x,y+\Delta y),(\Delta x,\Delta y)\in \{-i_{x},\ldots ,i_{x}\}\times \{-i_{y},\ldots ,i_{y}\}\}$$

**Figure 3:**
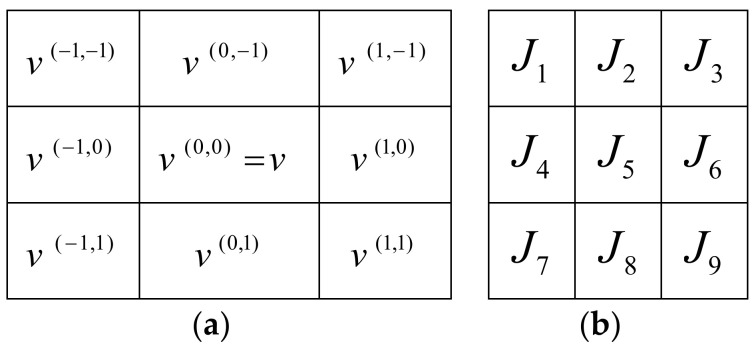
Motion Vectors (MVs) and their corresponding cost functions in a 3 × 3 neighborhood. (**a**) Motion Vectors; (**b**) cost functions.

These MVs and their corresponding cost functions in a 3 × 3 neighborhood are displayed in Figure 3.

**Figure 4:**
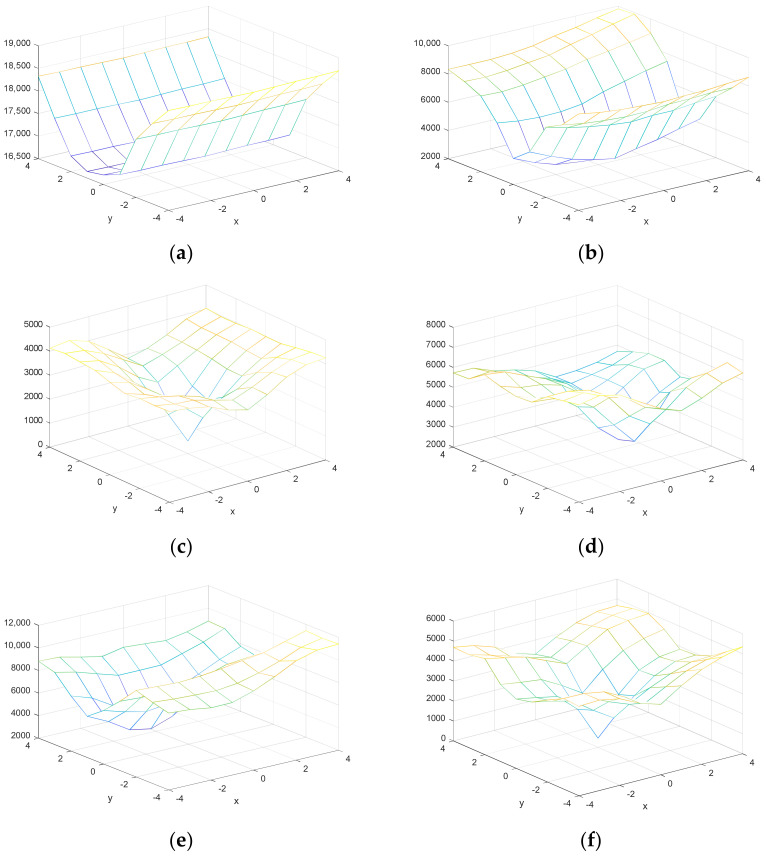
Sample cost function, first row for cover video and second row for stego video. (**a**) non-local optimal in (0,0); (**b**) non-local optimal in (0,0); (**c**) local optimal in (0,0); (**d**) non-local optimal in (0,0); (**e**) non-local optimal in (0,0); (**f**) local optimal in (0,0).

As observed in Figure 4, the sample cost function for cover and stego video in a 9 × 9 neighborhood.

From mentioned above and shown in Figure 4, the effect of quantification on the video cost function is obvious and a number of MVs are not local in their neighborhood without carrying a secret message.

The cost function can be obtained with different distortion functions, here we consider two common cost functions and extract the properties.

(14) J=SAD+λR

(15) JT=SATD+λR

Thus, the distinguishing features between the two classes of cover and stego were introduced using the following equations. These features are not sensitive to low variations of the cost function of each MV from the local minimum, which is often due to quantization and reveal the changes resulting from the insertion of the hidden message.

(16) $$f(k)=\frac{1}{N_{block}}\sum_{i=1}^{N_{block}}w_{i}.{\left(\frac{J_{k}^{i}-J_{5}^{i}}{J_{k}^{i}}\right)}^{2}.\;\delta (k-m_{i}),k=1,2,3,4,6,7,8,9$$

(17) $$f(5)=\frac{1}{N_{block}}\sum_{i=1}^{N_{block}}w_{i}.\delta (5-m_{i})$$

where $J_{k}^{i}$ is a *J* cost function for the *i*th block positioned in the *k*th position of the neighborhood and, $\delta (k-m_{i})=1$ if $k=m_{i}$, otherwise is 0 and $m_{i}=\arg\min\{J_{k}^{i}|k=1,\ldots ,9\}$.

Therefore, these 9 features are extracted from J and 9 other features also extracted from JT are as follow:

(18) $$f(k+9)=\frac{1}{N_{block}}\sum_{i=1}^{N_{block}}w_{i}.{\left(\frac{JT_{k}^{i}-JT_{5}^{i}}{JT_{k}^{i}}\right)}^{2}.\delta (k-mt_{i}),k=1,2,3,4,6,7,8,9$$

(19) $$f(14)=\frac{1}{N_{block}}\sum_{i=1}^{N_{block}}w_{i}.\delta (5-mt_{i})$$

where $JT_{k}^{i}$ is a *JT* cost function for the *i*th block positioned in the *k*th position of neighborhood and, $mt_{i}=\arg\min\{JT_{k}^{i}|k=1,\ldots ,9\}$.

**Figure 5:**
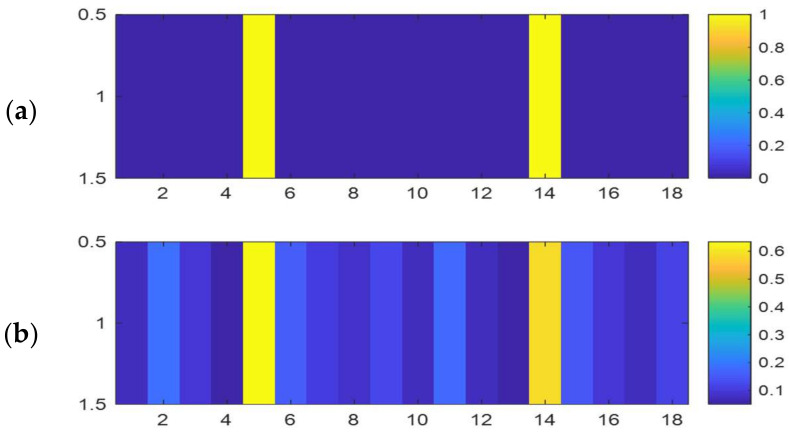
Sample feature vector extracted: (**a**) cover video; (**b**) stego video.

These 18 features are used for the SVM classification. Finally, the features are normalized. These features are shown for a cover and stego video. As seen in the Figure 5, for a cover video, the probability that the cost function of each vector is locally minimized is greater than its corresponding value in the stego video. 

Finally, classification is performed by using SVM classifier with the Gaussian kernel [35], then by 20% cross-validation on the grid space ($C\in \{2^{1},\ldots ,2^{15}\}$ and $\gamma \in \{2^{0},\ldots ,2^{-15}\}$), penalty parameter *C* and kernel parameter *γ* are optimized.

In the proposed method, by using the soft cluster (fuzzy cluster), the effect of none of the blocks is completely ignored and the blocks with even low texture also affect the final decision making. Only non-textured blocks have little weight in final decision-making, which means the extensive probability of these blocks is very limited in natural videos. Also, steganography methods often select the candidate blocks from all blocks, depending on the magnitude of the motion vector or the prediction error. In this case, the selection probability of the areas with only non-textured blocks are very low. For example, blocks that contain some natural landscapes like trees, grass, snow, sea waves and so forth, with lots of textures, are often used for steganography because of their high prediction error or high magnitude of motion vector. In the proposed method, these blocks with their large number of textures have more weight in decision making.

## 4. Computer Validation

In this section, various tests have been used for evaluating the proposed method. 284 uncompressed video sequences are used to create a cover and stego compressed video, which are downloaded from the Internet. All videos are CIF-resolution (352 × 288) and their color sampling is YUV 4:2:0. Three steganography methods are applied to the raw video during encoding to obtain various stego videos, to evaluate the proposed algorithm and other prominent steganalytic algorithms. At least one method has been selected from each generation of steganography methods. The first method is Aly’s method [4] (denoted by Aly’s), the second method is presented in Reference [6] (denoted by Cao’s) and the third method is proposed in method [13] (denoted by X.H’s).

Our method is compared with the three other steganalysis. One proposed in Reference [11] (denoted by Aoso), another presented in Reference [18] (denoted by IMVRB) and the last proposed by Zhang’s [3] (denoted by NPE). In comparison the proposed method against [3,11,18], the embedding capacity is calculated by the ratio of corrupted MV to total number of MVs (CMVR) in each frame. It is worth mentioning that all of the above-mentioned algorithms are performed in JM19.0 software.

Different ME methods were used for showing the performance of the proposed algorithm against fast and non-full search for considering the effect of different ME methods. ME is a fixed DIA (Diamond Search) [36] and HEX (Hexagon-based Search) [37].

Detection accuracy is expressed by the ratio of the number of correct detections of cover and stego videos to the total number of videos. Thus, this detection accuracy can be computed as [38]:

(20) $$\mathrm{accuracy}=\frac{\mathrm{tp}+\mathrm{tn}}{\mathrm{tp}+\mathrm{fp}+\mathrm{tn}+\mathrm{fn}}\times 100$$

where tp is the total number of correctly classified stego videos, tn is the total number of correctly classified cover videos, fp is the total number of incorrectly classified cover videos and fn is the total number of incorrectly classified stego videos.

For each simulation campaign, 60% of videos for training and 40% for testing were used. All Simulation is iterated 10 times and the average results are presented in the next.

### 4.1. Simulation 1

**Figure 6:**
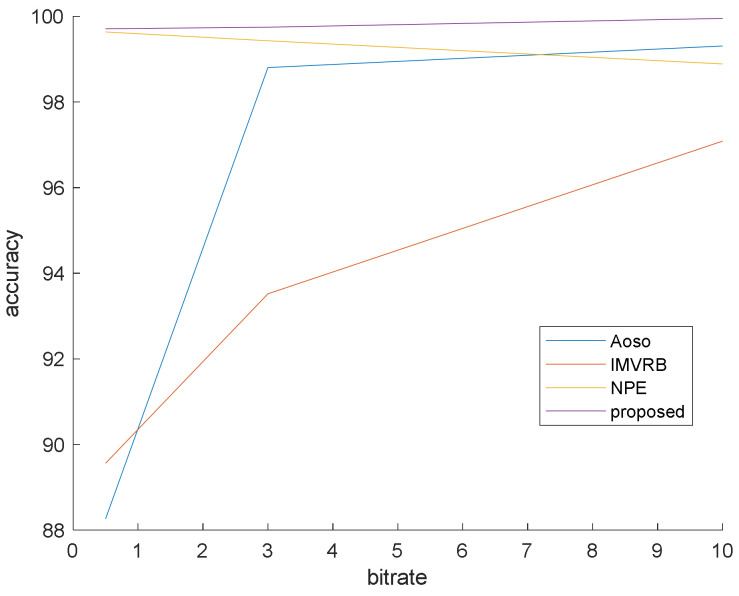
Steganalysis detection accuracy against Aly’s steganography.

**Figure 7:**
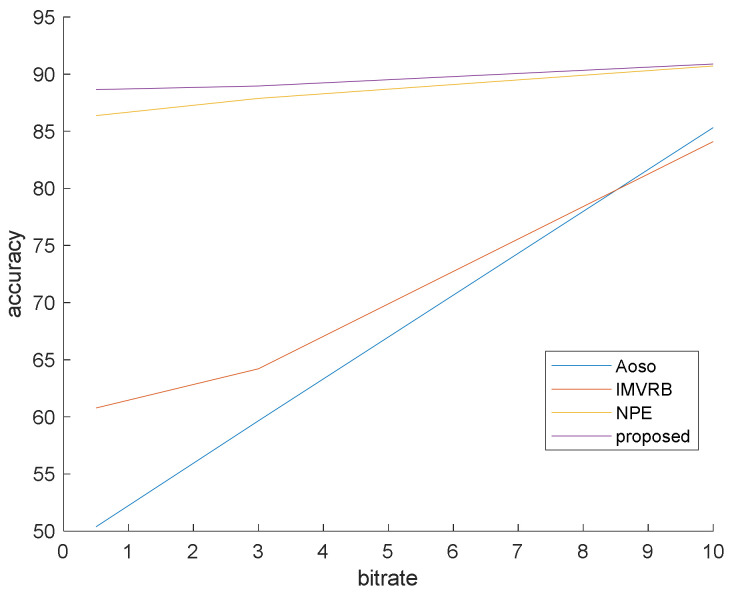
Steganalysis detection accuracy against Cao’s steganography.

**Figure 8:**
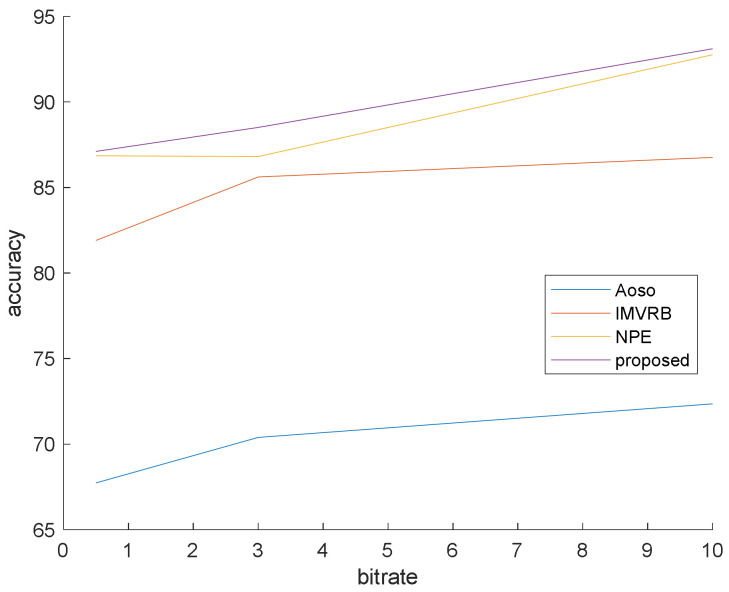
Steganalysis detection accuracy against Cao’s steganography.

**Figure 9:**
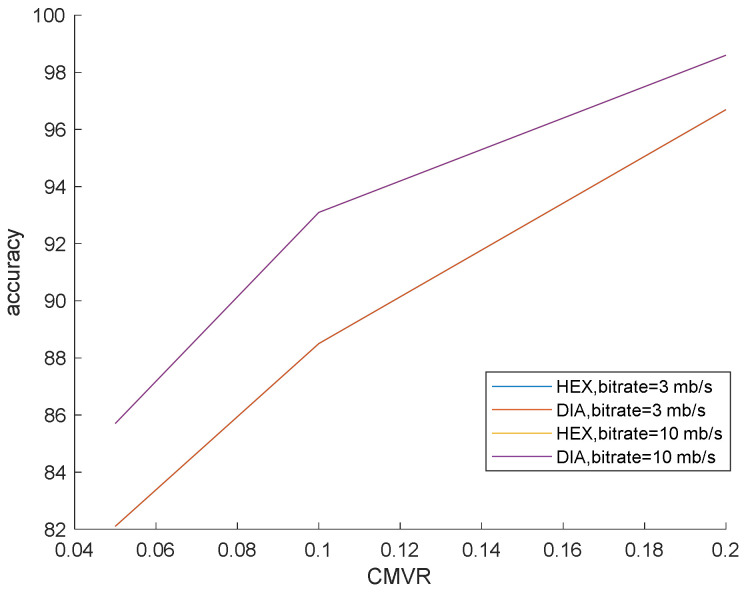
Steganalysis detection accuracy for different ME methods and bit rates against Cao’s steganography.

This simulation is prepared to validate that our method surpasses the famous preceding works. In this simulation, the bit rates are set at 0.5, 3 and 10 mb/s. Various bit rates are utilized to demonstrate the effect of quantization and lossy compression on the steganalysis features and their performance. The results are shown in Figure 6, Figure 7 and Figure 8 with CMVR = 0.1. From these figures, it is declared that by decreasing the bit rate, the detection accuracy of Aoso steganalyser decreases. Additionally, this method neglects the effects of number of bits in cost function and extracts features from the SAD instead of cost function. The IMVRB just used the recompression technique and intentioned in properties of reconstructing the ME equations. As a result, its detection accuracy is lower than all other methods against Aly’s steganography which changes the LSBs of MVs. The result changes according to ME methods are negligible for all steganalysers. On the other hand, all of them are resistant to the ME methods. Figure 9 illustrates the robustness of our method against the ME method.

By eliminating the negligible alterations of the cost function, the proposed algorithm can preserve detection accuracy over the bit rate variations and by considering regional feature (texture), it can obtain better result than others. These regional features weighted the blocks to participate in the final decision and did not use the same weight for all blocks as in other methods.

For a more complete comparison, Table 1 shows the comparison between the proposed method and the Arijit method [19] by ROC (receiver operating characteristic curve) characteristics. 40 and 60 bits per frame and full embedding rate apply to Aly’s steganography method to obtain stego videos. Then, Performance of steganalysers is measured. The detection accuracy and the area under the ROC curve (AUC) computed and reported in Table 1. 

### 4.2. Simulation 2

This situation in steganalysis is identified as the Cover Source Mismatch (CSM). CSM refers to simulation that the steganalysis classifier trained on one cover video source and it is utilized to test videos from a different source. In this simulation, the detection accuracy is adversely affected because of the discrepancy between both video sources. Results are represented in Table 2.

## 5. Conclusions

Videos with various bit rates, textures and movements need special steganalysis. Previous algorithms for the analysis of motion vectors were not sufficient enough to maintain intrinsic features of a desired video. Therefore, it is necessary to have a superior method to consider the weight of each block to reach a final property of a video. In this paper, by using the intrinsic and statistical features from each video, the improvement of the performance has been analyzed according to a proposed steganalysis method based on the entropy definition. Results of this regional feature, obtained from all blocks, validated the effectiveness. Also, such features were obtained from local optimality of the cost function. By a combination of this feature with the intrinsic feature, the final feature is prepared. As it was expected this method overcomes other prior prominent methods and robust against bit rate variation. In future research, more attention can be devoted to the H.265 video standard. 

## Figures and Tables

**Table 1:** AUC and detection accuracy of proposed method vs [19] against Aly’s steganography.

Embedding Rate	Arijit [19]		Proposed Method	
	AUC	Accuracy	AUC	Accuracy
40 bpfs	0.93	85	0.999	99.8
60 bpfs	0.98	93	0.999	99.86
Full	1	99	1	99.9

**Table 2:** Cover Source Mismatch (CSM) simulation results.

Steganography	Steganalysis			
	Aoso	IMVRB	NPE	Proposed
Aly’s	67.25	66.39	78.98	79.45
Cao’s	51.27	64.08	71.79	71.05
X.H’s	52.56	65.12	72.04	73.74
